# Low sensitivity of the new FIGO classification system for electronic fetal monitoring to identify fetal acidosis in the second stage of labor

**DOI:** 10.1016/j.eurox.2020.100120

**Published:** 2020-11-25

**Authors:** Frida Ekengård, Monika Cardell, Andreas Herbst

**Affiliations:** Department of Obstetrics and Gynecology Skåne University Hospital, Institution of Clinical Sciences Lund University, Lund, Sweden

**Keywords:** CTG, cardiotocography, FIGO, International Federation of Gynecology and Obstetrics, FIGO-15, FIGO classification system from 2015, SWE-09, the Swedish classification guidelines of CTG from 2009, SWE-17, the Swedish classification guidelines of CTG from 2017, Fetal monitoring, Fetal heart rate, Cardiotocograpy, Delivery, Asphyxia

## Abstract

**Objective:**

In 2015, new FIGO guidelines for CTG interpretation were presented (FIGO-15). In 2017, the previous Swedish guidelines (SWE-09) were replaced with guidelines adapted to FIGOs (SWE-17). The performance of these three templates had not been scientifically evaluated before its clinical implementation. The objective of this study was to compare the sensitivity and specificity to detect fetal acidosis at birth using these three templates during the second stage of labor.

**Study design:**

This case-control study included 295 neonates with cord blood pH < 7.05 and 591 controls with pH ≥ 7.15, born 2012−2017. Tracings from the last 30−80 min of labor were classified independently by three assessors (midwives, residents and obstetricians), blinded to group and outcome.

**Results:**

The classification pathological using FIGO-15 had a sensitivity of 50 % and specificity of 88 % in detecting fetuses with acidosis. For SWE-17, the sensitivity was 62 % and the specificity 85 %. For SWE-09 the sensitivity was 87 % and the specificity 56 %.

By combining suspicious and pathological patterns the sensitivity for FIGO-15 increased to 97 %, and for SWE-17 to 83 %, whereas the specificity decreased to 23 % and 68 % respectively.

**Conclusions:**

The FIGO classification seemed to be insufficiently discriminative in the second stage of labor; most patterns in acidotic cases were classified as merely suspicious with this template, and the sensitivity of pathological patterns was low at 50 %. Combined pathological and suspicious patterns detected fetal acidosis at a specificity that was too low to be useful (23 %). SWE-09 showed the best ability to detect acidosis with pathological patterns (sensitivity 87 %). SWE-17 reached almost the same sensitivity (83 %) with the combination of suspicious and pathological patterns, and at a higher specificity (68 %).

## Highlights

• Cardiotocography interpretation guidelines evaluated during second stage of labor.
• Case-control study including neonates with cord artery acidosis at vaginal delivery.
• Low sensitivity of FIGO intrapartum monitoring guidelines to detect acidosis.
• The Swedish 2009 template had a high sensitivity.
• The Swedish 2017 template had a high sensitivity with cut-off set at suspicious.

## 1 Introduction

Surveillance with cardiotocography (CTG) aims to detect signs of fetal hypoxia, enabling intervention before asphyxia occurs. Randomised studies from 1976 to 1993 comparing CTG with intermittent auscultation indicated that CTG monitoring lowered the incidence of neonatal seizures [1,2] but only one study showed a reduction of perinatal mortality [3]. Since then, the knowledge about CTG interpretation has grown [4].

In 1987 FIGO presented the first international guidelines of CTG [5], that was modified to different guidelines over the years. The systems differ in many aspects [6], leading to different classifications of the same tracings [7], and likely to different clinical decisions. In Sweden a national classification system for CTG interpretation, modified from FIGO-1987 and from the STAN template from 2007, was in use 2009–2016, SWE-09 [8].

In 2010 Ayres-De-Campos and Bernardes concluded that the FIGO guidelines from 1987 had limitations and called for a simpler and more objective guideline [6]. Not having an internationally accepted guideline was thought to lower the effectiveness of CTG [6]. In 2015 a new guideline and classification template on intrapartum fetal monitoring was introduced, FIGO-15 [9]. In Sweden, FIGO-15 was adjusted to SWE-17(10), replacing SWE-09.

CTG patterns are often markedly different in the first and second stage of labor. The second stage is the period of highest risk of hypoxia, and the fetus is affected by the higher intrauterine pressure [11,12].

This study was undertaken to evaluate the sensitivity and specificity for the three templates in detecting hypoxia during the second stage of labor, as indicated by acidosis at birth after vaginal delivery or after cesarean section in the second stage of labor.

The primary objective of this study was to compare the sensitivity and specificity of FIGO-15, SWE-09 and SWE-17, in identifying cases with acidosis at birth in the second stage of labor.

## 2 Materials and methods

This is a retrospective case control study including neonates with acidemia (case group) defined as cord artery or cord vein pH < 7.05, and controls defined as neonates with a cord artery and cord vein pH ≥ 7.15 and Apgar scores of 9 or 10 at five and ten minutes, all with CTG monitoring during the second stage of labor.

A power calculation estimated that 215 cases were needed to detect a difference in sensitivity between 80 % and 90 %, with 80 % power and a p-value of 0.05. To detect a difference in specificity between 60 % and 70 %, 386 controls were needed.

Cases and controls were collected from births at Skåne University Hospital in Malmö and Lund April 23^d^2013 – October 31st 2017, and Helsingborg Hospital March 13th2012 – December 31st 2016. Inclusion criteria for both groups were singleton pregnancy and an available CTG tracing of at least 30 min, ending at vaginal birth or within 30 min of second stage cesarean delivery. The case group consisted of newborns with cord artery or cord vein pH < 7.05 after vaginal birth or cesarean delivery in the second stage of labor. As controls, the first two neonates born consecutively after each case at the same hospital fulfilling the inclusion criteria above, except for acidosis, and who had both cord artery and cord vein pH ≥ 7.15 and at least 0.02 apart and Apgar scores 9 or 10 at five and ten minutes, were included.

In the international guidelines [9], preterm birth is not mentioned, but in the Swedish national guidelines [10], it is stated that after 34 weeks, guidelines for full term are used. We therefore excluded births prior to 34 full weeks.

Clinical data was gathered from patient files and computerized CTG tracings were evaluated. The last 30 min, and when available up to 80 min, of the tracings before birth were assessed. The tracings were anonymized and randomized.

The interpretations of the CTG tracings were performed independently by three professionals, representing trained obstetric staff (midwives, residents and obstetricians) with different levels in experience of electronic fetal monitoring from their daily work. All had performed educational programs including both the previous and the current classification templates. Each of the 886 traces were assessed by 3 of totally 21 interpreters. Each interpreter received a portfolio with tracings, information about the study and classification forms. The only additional clinical information provided was that it was a singleton pregnancy ending in vaginal birth or cesarean delivery in the second stage of labor. The graphic of the tracings was 1 cm/min.

Each interpreter completed a form including the assessment of all the variables relevant for classification. This was done twice; in 2017 with a protocol for SWE-09 when it was in clinical use, and in 2018 with a protocol for FIGO-15 and SWE-17 when the interpreters had been re-educated and used the SWE-17 in clinical practice. The description of all the included variables by each interpreter were then used to classify strictly according to each template to classification normal, suspicious or pathological.

The final classification was composed of the three professionals’ assessments of the variables in each trace, and the classification for each interpreter for each template, representing the majority assessment of obstetric staff with different experience. If at least two out of three assessments agreed, that was the final classification. If all three classifications differed, a fourth assessor, an experienced obstetrician, also classified the trace, so that a final judgement was attained for all traces.

### 2.1 Outcomes

The main outcome was the sensitivity for the classification pathological to identify cases with acidosis at birth, and the specificity for the classification normal or suspicious together to rule out acidosis. The classification preterminal using SWE-09 was regarded as pathological in the final analyses. The interpretation suspicious mandates continued surveillance combined with additional active management to correct reversible causes and to evaluate the fetal condition in all three templates. Therefore, we also evaluated the sensitivity of suspicious and pathological patterns combined in identifying acidosis, and the specificity for the classification normal to rule out acidosis.

### 2.2 Statistical analyses

The information was gathered in Stat View® computer software. The sensitivity and specificity with 95 % CI for the final classification was calculated for the three classification systems, using www.sample-size.net/confidence-interval-proportion provided by UCSF. The chi-square test was used to determine if there was a significant difference in sensitivity or specificity between classification systems, and a p-value < 0.05 was considered statistically significant.

### 2.3 Ethical approval

Ethical approval was obtained from the Regional Ethical Review Board in Lund, Dnr 2016/371, 2016-05-24.

## 3 Results

**Fig. 1:**
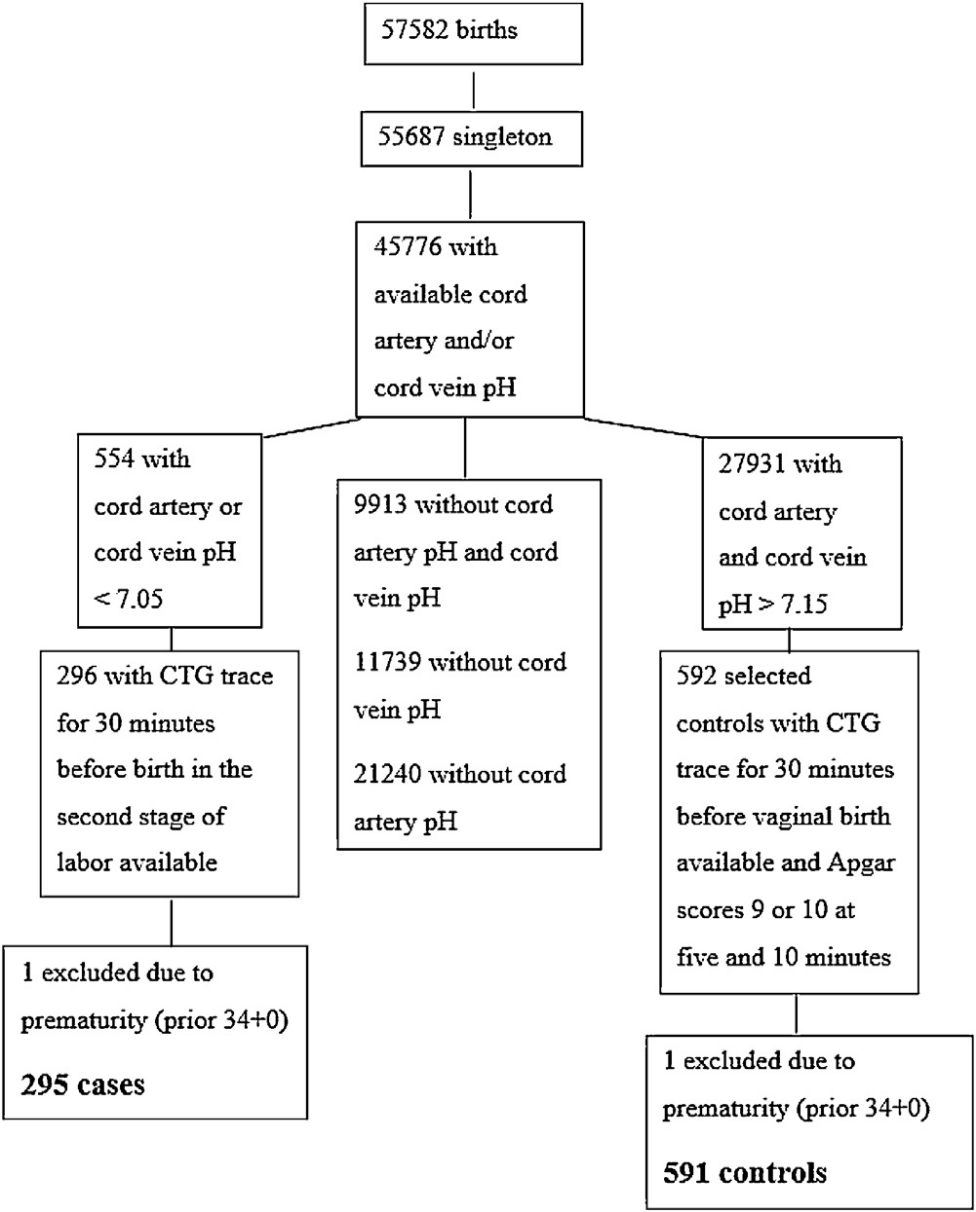
Retrieval of cases and controls.

During the study period 57,582 neonates were born at the two hospitals. A total of 296 cases and 592 controls fulfilling the inclusion criteria were included. One case and one control were excluded due to birth before 34 gestational weeks, leaving 295 cases and 591 controls in the study (Fig. 1). Background data are summarized in Table 1.

**Table 1:** Summary of background data of cases and controls.

	Cases total n (%)	Controls total n (%)
Total	295	591
Primipara	190 (64.4)	297 (50.3)
Instrumental delivery	88 (29.8)	33 (5.6)
Cesarean delivery	24 (8.1)	0
Shoulder dystocia	10 (3.4)	0
Preterm birth	8 (2.7)	18 (3.0)
Post-term birth	21 (7.1)	33 (5.6)
Birthweight < 2.5 kg	3 (1.0)	8 (1.4)
Birthweight > 4.5 kg	10 (3.4)	13 (2.2)
Breech	2 (0.7)	1 (0.2)
Epidural	125 (42.4)	177 (29.9)
Fever/infection	5 (1.7)	3 (0.5)
Meconium stained amniotic fluid	65 (22.0)	106 (17.9)
Diabetes	14 (4.7)	14 (2.4)
Preeclampsia	10 (3.4)	12 (2.0)
BMI <25	181 (61.4)	361 (61.1)
BMI > 30	30 (10.1)	65 (11.0)
Smoking	17 (5.8)	41 (6.9)
Female fetus	133 (45.1)	307 (51.9)
5-minute Apgar score <7	34 (11.5)	0
Base excess < -12	76 (25.8)	2 (0.3)

Rates of agreement between the classifications determined by the assessment of the included variables of the three interpreters are shown for cases and controls in Table 2. For classification of cases, the agreement was highest for SWE-09, whereas for controls agreement was higher for FIGO-15 and SWE-17.

**Table 2:** Agreement for classifications based on the assessments of three independent interpreters of cases and controls with the three templates.

	SWE-09 n (%)	SWE-17 n (%)	FIGO-15 n (%)
Cases			
All three agreed	213 (72.2)	128 (43.4)	147 (49.8)
Two of three agreed	78 (26.4)	147 (49.8)	141 (47.8)
None agreed*	4 (1.4)	20 (6.8)	7 (2.4)

Controls			
All three agreed	236 (39.9)	297 (50.3)	311 (52.6)
Two of three agreed	318 (53.8)	263 (44.5)	272 (46.0)
None agreed*	37 (6.3)	31 (5.2)	8 (1.4)

* In these cases, a 4th interpreter (experienced obstetrician) was added to attain a final classification.

The result of the final classifications is summarized in Table 3, and the sensitivity and specificity for the different templates in Table 4. The sensitivity for the classification pathological to identify cases with acidosis differed significantly between the classifications systems: 87.1 % for SWE-09, 62.0 % for SWE-17 and 50.2 % for the FIGO-15 classification system. The corresponding specificity was higher for FIGO-15 (87.5 %) and SWE-17 (84.8 %) than for SWE-09 (55.5 %).

**Table 3:** Summary of classifications of CTG tracings in cases, and controls with the three templates.

	SWE-09 n (%)	SWE-17 n (%)	FIGO-15 n (%)
Cases	295	295	295
Normal	7 (2.4)	49 (16.6)	10 (3.4)
Suspicious	31 (10.5)	63 (21.4)	137 (46.4)
Pathological	257 (87.1)	183 (62.0)	148 (50.2)
*Of which preterminal*	*33(11.2)*		

Controls	591	591	591
Normal	167 (28.3)	400 (67.7)	133 (22.5)
Suspicious	161 (27.2)	101 (17.1)	384 (65.0)
Pathological	263 (44.5)	90 (15.2)	74 (12.5)
*Of which preterminal*	*3 (0.5)*

**Table 4:** Comparison of sensitivity and specificity for the different templates to identify neonatal acidosis in the second stage of labor.

	Sensitivity, % (95 % CI)	Specificity, % (95 % CI)
Classification Pathological		
FIGO-15	50.2 (44.3−56.0)^1^	87.5 (84.5−90.0)
SWE-17	62.0 (56.2−67.6)^1^	84.8 (81.6−87.6)
SWE-09*	87.1 (82.8−90.7)^1^	55.5 (51.4−59.6)^1^

Classification Pathological + suspicious		
FIGO-15	96.6 (93.9−98.4)	22.5 (19.2−26.1)^2^
SWE-17	83.4 (78.6−87.5)^1^	67.7 (63.5−71.4)^1^
SWE-09*	97.6 (95.2−99.0)	28.3 (24.7−32.1)^2^

* Including patterns classified as preterminal.

1 Differs significantly from each of the other two (p < 0.01).

2 Differs significantly from each of the other two (p < 0.05).

When combining suspicious and pathological patterns the sensitivity for SWE-17 increased to 83.4 %, which was not significantly lower than the sensitivity for pathological patterns with SWE-09 (p = 0.26), whereas the specificity at 67.7 % was significantly higher than for pathological patterns with SWE-09 (p < 0.001). For the FIGO-15, combing suspicious and pathological patterns also lead to a high sensitivity (96.6 %), but the specificity declined to a very low level (22.5 %).

## 4 Discussion

### 4.1 Interpretation of the main results

In this study we found that during the second stage of labor the FIGO-15 template had the lowest sensitivity, 50.2 %, to detect fetal acidosis when the cut-off was pathological patterns. When the cut-off was suspicious patterns, it had the lowest specificity, 22.5 %. The template lead to a high rate of patterns classified as suspicious in cases (46.4 %) as well as in controls (65.0 %).

The SWE-09 template had the highest sensitivity to detect acidotic fetuses (87.1 %), whereas the specificity was low (55.5 %). Combining pathological and suspicious increased the sensitivity to 97.6 % with a concomitant decrease of specificity to 28.3 %.

The SWE-17, modelled on the FIGO-15, had a sensitivity of 62.0 % for pathological patterns. When the cut-off was set at suspicious patterns the sensitivity was 83.4 %. This is similar as for pathological patterns with SWE-09, but the specificity was higher, 67.7 %.

We consider that the safety of SWE-17 and SWE-09 was similar, if also suspicious patterns are acted upon with SWE-17. Acting does not always have to be to deliver, but may include diagnostic measures (fetal blood sampling) as well as other therapeutic measures (alleviating oxytocin overstimulation). The FIGO-15 classification results in too many suspicious tracings to be discriminative. The sensitivity of the classification pathological for FIGO-15 was low, and the specificity with a cut-off at suspicious was also low. This finding raises doubts concerning the validity in clinical practice.

It is not clear to us why the interpretation using the SWE-17 template resulted in a higher rate of normal classification in acidotic fetuses (16.6 %) compared to both SWE-09 (2.4 %) and FIGO-15 (3.4 %). The main difference between the SWE-17 compared to the SWE-09 ad FIGO-15 is the definition of decelerations. This merits further analysis.

### 4.2 Comparison of the present results with previous studies

A few previous studies have compared different CTG interpretation templates [13,14] and many studies have analyzed the association between specific CTG patterns and acidemia [15,16]. Coletta et al. evaluated a 3-tier and a 5-tier classification system in a study including 30 cases with pH < 7.00 and 24 controls with pH > 7.20 [17]. They found a 79 % sensitivity and a 100 % specificity of the two worst categories in the 5-tier system to detect acidosis at birth, whereas the 3-tier system, that was similar to FIGO-15, had a low sensitivity (12.5 %) since most cases and controls were categorized as category 2. They did not present confidence intervals for sensitivity and specificity and the number of cases and controls were limited.

Bhatia et al. compared FIGO-15 with the NICE guidelines from 2007 and 2014 [18]. They found that FIGO-15 offered favorable agreement scores, was easy to use and lead to a moderate rate of interventions. However, that study did not address the validity of the classification.

Olofsson et al. compared the STAN classification system from 2007 (similar to SWE-09) with FIGO-15. They found that the two systems classified tracings differently [19] and that the FIGO-15 had a lower sensitivity (43 %) than the STAN template (73 %) [20]. Martí Gamboa et al. [21] compared the FIGO-15 classification form with a 5-tier classification [22], in 102 cases with pH ≤7.10 and base deficit > 8 mmol/l, and 100 controls. The FIGO classification had a sensitivity of 43.6 %, and a specificity of 82.5 %, and the 5-tier system a sensitivity of 36 %, and 88 % specificity for acidosis at birth. The present study confirms the results of these studies, with a low sensitivity for the FIGO classification system to detect fetal acidosis.

### 4.3 Strengths and weaknesses of study design and methods

The case group was defined as neonates born with a pH < 7.05. Jonsson et al. reported pH < 7.05 at birth as a useful variable for quality control of management of the second-stage of labor [23]. A cord artery pH of 7.01–7.05 ha s been associated with a 10-fold risk of encephalopathy with early neonatal seizures [24]. Thus, it is desirable for our monitoring methods to detect hypoxia resulting in this degree of acidosis.

Each trace was interpreted by at least three different individuals, and the final classification was that of the majority (or two out of four). This might result in a higher sensitivity and specificity than if only one person assesses a trace. We chose the design with triple interpretation of each trace by clinicians with different experience both to reflect clinical practice and to achieve more accurate interpretations of the fetal heart rate parameters than from assessments of a single individual.

The interpreters in the study were blinded to outcome, which is necessary to avoid ascertainment bias [25,26]. In the clinical management of labor, CTG is just one part of the management, but since our purpose was to assess the CTG templates as such, and not clinical management, we considered it proper to leave the interpreters blind for clinical data, minimizing the risk of bias.

To eliminate bias caused by different experience of the different classification systems, the classification of the different fetal heart rate parameters (heart rate, decelerations etc.) were systematically transformed to classifications normal, suspicious or pathological according to each classification form by one of the authors.

### 4.4 Concluding discussion

The results of the study indicate that we are in dire need for improvement of interpretation protocols to ensure safe labor care. The purpose of intrapartum fetal monitoring is to detect fetal hypoxia in time to prevent asphyxia. With a low sensitivity of monitoring, fetal asphyxia may not be possible to avoid, whereas a low specificity may lead to unnecessary interventions. We consider a high sensitivity of CTG to be more important than a high specificity, since identification of fetuses at risk is indispensable if asphyxia should be avoidable, and since secondary diagnostic methods can be used to improve specificity.

Further studies are needed to analyze how differences between the different parameters in the templates affect sensitivity and specificity. Improvements of SWE-09 to increase specificity or of SWE-17 to increase sensitivity might be possible, but before introducing new templates for fetal monitoring in clinical practice, studies of the validity of such templates should be performed.

## 5 Conclusion

We consider the FIGO-15 classification to be too restrictive in classifying tracings as pathological, and too liberal in the classification of merely suspicious tracings, to properly be able to discriminate between fetuses with and without acidosis. The sensitivity of the classification “pathological” to detect acidosis was low and insufficient for safe surveillance. The classification “suspicious” however, implied fetal acidosis with too low specificity to be clinically useful and to require clinical action in all cases of a suspicious trace. SWE-17 provided the best combination of sensitivity (83.4 %) and specificity (67.7 %), if the cut-off for indicating fetal acidosis was set at a suspicious pattern, whereas SWE-09 provided the best sensitivity (87.1 %) for pathological patterns and had the lowest false negative rate of tracings classified as normal in cases with acidosis (2.4 %).

## Funding

This work was supported by research grants from Region Skåne and funding from LÖF, the national Swedish patient insurance company. The funders played no role in planning or conducting the research or writing of the paper.

## Declaration of Competing Interest

The authors declare that they have no competing financial interest that have influenced the work reported in this paper. Andreas Herbst has contributed in the development of the Swedish classification systems from 2009 and 2017.

## References

[1] MacDonald D., Grant A., Sheridan-Pereira M., Boylan P., Chalmers I. (1985). The Dublin randomized controlled trial of intrapartum fetal heart rate monitoring. Am J Obstet Gynecol.

[2] Alfirevic Z., Devane D., Gyte G.M., Cuthbert A. (2017). Continuous cardiotocography (CTG) as a form of electronic fetal monitoring (EFM) for fetal assessment during labour. Cochrane Database Syst Rev.

[3] Vintzileos A.M., Antsaklis A., Varvarigos I., Papas C., Sofatzis I., Montgomery J.T. (1993). A randomized trial of intrapartum electronic fetal heart rate monitoring versus intermittent auscultation. Obstet Gynecol.

[4] Vintzileos A.M., Nochimson D.J., Antsaklis A., Varvarigos I., Guzman E.R., Knuppel R.A. (1995). Comparison of intrapartum electronic fetal heart rate monitoring versus intermittent auscultation in detecting fetal acidemia at birth. Am J Obstet Gynecol.

[5] Rooth G., Huch A., Huch R. (1987). Guidelines for the use of fetal monitoring. Int J Gynaecol Obstet.

[6] Ayres-de-Campos D., Bernardes J. (2010). Twenty-five years after the FIGO guidelines for the use of fetal monitoring: time for a simplified approach?. Int J Gynaecol Obstet.

[7] Santo S., Ayres-de-Campos D., Costa-Santos C., Schnettler W., Ugwumadu A., Da Graca L.M. (2017). Agreement and accuracy using the FIGO, ACOG and NICE cardiotocography interpretation guidelines. Acta Obstet Gynecol Scand.

[8] SFOG (2009). CTG kort slutversion Sweden: SFOG. https://www.sfog.se/media/17094/ctg_kort__slutversion.pdf.

[9] Ayres-de-Campos D., Spong C.Y., Chandraharan E. (2015). FIGO consensus guidelines on intrapartum fetal monitoring: cardiotocography. Int J Gynaecol Obstet.

[10] SFOG (2017). CTG och fosterövervakning Sweden: CTG-utbildning. http://ctgutbildning.se.

[11] Pinas A., Chandraharan E. (2016). Continuous cardiotocography during labour: analysis, classification and management. Best Pract Res Clin Obstet Gynaecol.

[12] Ugwumadu A. (2013). Understanding cardiotocographic patterns associated with intrapartum fetal hypoxia and neurologic injury. Best Pract Res Clin Obstet Gynaecol.

[13] Cahill A.G., Roehl K.A., Odibo A.O., Macones G.A. (2012). Association and prediction of neonatal acidemia. Am J Obstet Gynecol.

[14] Elliott C., Warrick P.A., Graham E., Hamilton E.F. (2010). Graded classification of fetal heart rate tracings: association with neonatal metabolic acidosis and neurologic morbidity. Am J Obstet Gynecol.

[15] Parer J.T., King T., Flanders S., Fox M., Kilpatrick S.J. (2006). Fetal acidemia and electronic fetal heart rate patterns: is there evidence of an association?. J Maternal-Fetal & Neonatal Med.

[16] Holzmann M., Wretler S., Cnattingius S., Nordstrom L. (2015). Cardiotocography patterns and risk of intrapartum fetal acidemia. J Perinat Med.

[17] Coletta J., Murphy E., Rubeo Z., Gyamfi-Bannerman C. (2012). The 5-tier system of assessing fetal heart rate tracings is superior to the 3-tier system in identifying fetal acidemia. Am J Obstet Gynecol.

[18] Bhatia M., Mahtani K.R., Nunan D., Reddy A. (2017). A cross-sectional comparison of three guidelines for intrapartum cardiotocography. Int J Gynaecol Obstet.

[19] Olofsson P., Noren H., Carlsson A. (2018). New FIGO and Swedish intrapartum cardiotocography classification systems incorporated in the fetal ECG ST analysis (STAN) interpretation algorithm: agreements and discrepancies in cardiotocography classification and evaluation of significant ST events. Acta Obstet Gynecol Scand.

[20] Olofsson P., Noren H., Carlsson A., Rosen K.G. (2018). Identifying newborns with umbilical cord blood metabolic acidosis by intrapartum cardiotography combined with fetal ECG ST analysis (STAN): comparison of the new and old FIGO systems to classify cardiotocograms. J Maternal-Fetal & Neonatal Med.

[21] Marti Gamboa S., Gimenez O.R., Mancho J.P., Moros M.L., Sada J.R., Mateo S.C. (2017). Diagnostic accuracy of the FIGO and the 5-Tier fetal heart rate classification systems in the detection of neonatal acidemia. Am J Perinatol.

[22] Parer J.T., Ikeda T. (2007). A framework for standardized management of intrapartum fetal heart rate patterns. Am J Obstet Gynecol.

[23] Jonsson M., Norden Lindeberg S., Ostlund I., Hanson U. (2013). Acidemia at birth in the vigorous infant as a trigger incident to assess intrapartum care with regard to CTG patterns. J Maternal-Fetal & Neonatal Med.

[24] Yeh P., Emary K., Impey L. (2012). The relationship between umbilical cord arterial pH and serious adverse neonatal outcome: analysis of 51,519 consecutive validated samples. BJOG.

[25] Reif P., Schott S., Boyon C., Richter J., Kavsek G., Timoh K.N. (2016). Does knowledge of fetal outcome influence the interpretation of intrapartum cardiotocography and subsequent clinical management? A multicentre European study. BJOG.

[26] Ayres-de-Campos D., Arteiro D., Costa-Santos C., Bernardes J. (2011). Knowledge of adverse neonatal outcome alters clinicians’ interpretation of the intrapartum cardiotocograph. BJOG.

